# 2145 Drug formulation strategies: A vital but nearly invisible component in translational education

**DOI:** 10.1017/cts.2018.205

**Published:** 2018-11-21

**Authors:** Robert B. MacArthur, Roger Vaughan, Barry S. Coller

**Affiliations:** Rockefeller University

## Abstract

OBJECTIVES/SPECIFIC AIMS: To develop a KL2 curriculum on the science and art of drug formulation. METHODS/STUDY POPULATION: Develop training materials for KL2 scholars that outline the art of formulation development. Materials will include syllabi, reading materials, and course work. RESULTS/ANTICIPATED RESULTS: This will enhance the training of KL2 scholars by incorporating formulation development concepts into their human health enhancing research projects. DISCUSSION/SIGNIFICANCE OF IMPACT: For new chemical entities, formulation goals must be realistic and move along in a step-wise manner from the laboratory bench, through toxicology studies, and on to Phase 1 studies. By training scholars in phase-specific formulation goals, their interactions with funding agencies, formulation scientists, and regulators will be more efficient, productive, and successful. For those scholars who are working to improve existing treatments, introducing the concept of formulation improvements that can create new indications, or improve efficacy, safety and patient compliance will open up more possibilities for creative product development.

